# The need to strengthen laboratory leadership, systems, and networks to enhance outbreak detection and resilience in Africa: proceedings of a regional workshop

**DOI:** 10.1186/s12919-026-00382-4

**Published:** 2026-06-09

**Authors:** Harris Onywera, Richard Njouom, Dorcas Waruguru Wanjohi, Jackson Peter Claver Mushumbusi, Abdou Moindze, Abebaw Kebede, Alisen Ayitewala, Ambele Eliah Mwafulango, Anne von Gottberg, Collins Kipngetich Tanui, Euniverse Shikaseba, Fahima Moosa, Felix Koukouikila-Koussounda, Ibrehima Guindo, Isaac Ssewanyana, Joseph Bitilinyu-Bangoh, Joseph Nyandwi, Kedibone Maria Ndlangisa, Klèma Marcel Koné, Mignon du Plessis, Mirriam Ethel Nyenje, Moussa M. Diagne, Natalie T. Mayet, Nebiyu Dereje, Noumou Yakhouba Keita, Ouindgueta Juste Isidore Bonkoungou, Placide Mbala, Prince Akil-Bandali, René Ghislain Essomba, Roch Fabien Niama, Roma Chilengi, Sarah Mwangi, Seydou Barro, Sikhulile Moyo, Susan N. Nabadda, Talkmore Maruta, Tonny Muyigi, Valentina J. Ngo Bitoungui, Umar Ahmad, Vindana Chibabhai, Winnie Kwamboka Mokaya, Youssouf Tadjidine, Sofonias K. Tessema, Jean Kaseya, Yenew Kebede Tebeje

**Affiliations:** 1 Africa Centres for Disease Control and Prevention (Africa CDC), Addis Ababa, Ethiopia; 2 Centre Pasteur du Cameroun (CPC), Yaoundé, Cameroon; 3 National Public Health Laboratory (NPHL), Dar Es Salaam, Tanzania; 4 Department of Disease Control, National Hospital Centre El Maarouf, Moroni, Union of the Comoros; 5 Central Public Health Laboratories (CPHL), National Health Laboratory and Diagnostic Services (NHLDS), Ministry of Health, Kampala, Uganda; 6 National Institute for Communicable Diseases (NICD), National Health Laboratory Service (NHLS), Johannesburg, South Africa; 7 Zambia National Public Health Institute (ZNPHI), Lusaka, Zambia; 8 National Public Health Laboratory (NPHL), Brazzaville, Republic of the Congo; 9 Institut National de Santé Publique (INSP), Bamako, Mali; 10 Public Health Institute of Malawi (PHIM), Ministry of Health, Lilongwe, Malawi; 11 Institut National de Santé Publique (INSP), Bujumbura, Burundi; 12 Faculty of Health Sciences, School of Pathology, University of the Witwatersrand, Johannesburg, South Africa; 13 Institut Pasteur de Dakar (IPD), Dakar, Senegal; 14 National Public Health Institute (NPHI), Ouagadougou, Burkina Faso; 15 Institut National de La Recherche Biomédicale (INRB), Kinshasa, Democratic Republic of Congo; 16 National Public Health Laboratory (NPHL), Yaoundé, Cameroon; 17 Botswana Harvard Health Partnership, Gaborone, Botswana; 18 Harvard T.H. Chan School of Public Health, Boston, USA; 19 Division of Medical Virology, Department of Pathology, Stellenbosch University, Cape Town, South Africa; 20 School of Allied Health Professions, University of Botswana, Gaborone, Botswana; 21 School of Health Systems and Public Health, University of Pretoria, Pretoria, South Africa; 22 African Society for Laboratory Medicine (ASLM), Addis Ababa, Ethiopia

**Keywords:** Laboratory leadership, Laboratory systems strengthening, Public health, Infectious disease outbreaks, Outbreak detection, Africa

## Abstract

Africa bears a disproportionate burden of infectious disease outbreaks and antimicrobial resistance, compounded by weaknesses in public health laboratory systems. In line with its mandate to safeguard public health, the Africa Centres for Disease Control and Prevention (Africa CDC) supports its Member States in strengthening public health laboratory capacity. However, gaps in laboratory leadership continue to threaten the resilience and sustainability of these systems. To address this need, Africa CDC convened a Laboratory Leadership Workshop under the Africa Pathogen Genomics Initiative (Africa PGI) – DETECT Project from 27–29 August 2025 in Mombasa, Kenya. The meeting brought together 24 heads of public health and reference laboratories and senior technical experts from 15 Member States. Combining expert presentations, country case studies, and roundtable discussions, participants examined system-level challenges affecting laboratory performance. Workshop proceedings were synthesized according to the predefined thematic areas that guided the workshop discussions, drawing on rapporteurs’ reports and validated through participant review. Key issues included overreliance on external funding, fragile procurement and supply chains, limited equipment servicing, weak sample referral networks, fragmented data systems, and inadequate contingency planning. Comparative country experiences showed that stronger domestic financing and institutionalized leadership structures were linked to greater operational continuity. Emerging innovations such as hybrid decentralization of diagnostic services and integrated sample referral networks were discussed, with success contingent on strengthened leadership in strategic planning, resource mobilization, and cross-sector coordination. The workshop identified cross-cutting priorities, including financial independence, sustainable equipment servicing, quality management, One Health-aligned digital interoperability, and leadership networks for building resilient systems. The meeting concluded that beyond infrastructure, Africa’s epidemic preparedness depends on empowered and committed leaders capable of overcoming challenges to steer resilient and sustainable laboratory systems.

## Introduction

Africa experiences a high and growing burden of public health events, with the WHO African Region reporting over 100 major emergencies annually, including outbreaks of Ebola virus disease (EVD), cholera, yellow fever, dengue, mpox [1], varicella [2, 3], and growing burden of antimicrobial resistance (AMR) [4, 5]. These events impose substantial strain on health systems, disrupt essential health services, increase morbidity and mortality, particularly among vulnerable populations, and negatively affect economic productivity and social cohesion [1, 6, 7]. The threat of emerging and re-emerging pathogens demands strengthened laboratory capacity, effective leadership, and coordinated surveillance networks. Recent outbreaks such as EVD in Uganda [8], Marburg in Tanzania [9], Rwanda [10], and Ethiopia [11], as well as cholera, now endemic in several African countries [12] highlight the urgent need for robust laboratory systems and regional coordination to improve diagnostic capacity and strengthen public health resilience.

In Africa, National Public Health Institutes (NPHIs) serve as the country’s technical and coordination pillar for protecting population health and ensuring health security, yet their establishment is uneven across the continent [13]. They monitor diseases and outbreaks, lead emergency preparedness and response, oversee public health laboratory networks, conduct applied research to inform policy, train the public health workforce, manage interoperable health information systems, promote disease prevention and healthy behaviours, provide evidence-based policy guidance, and, often under national legislation, coordinate cross-sectoral, regional, and international partnerships to strengthen essential public health functions and system resilience [13, 14]. Africa CDC is a continental autonomous public health agency of the African Union (AU) that supports the development and sustainability of NPHIs, including their laboratories, through policy guidance, technical assistance, infrastructure support, and regional coordination to strengthen core public health capacities across Member States. However, the effectiveness of NPHIs and laboratory systems in many African countries is affected by enormous challenges, such as insufficient leadership and governance, limited financial resources, inadequate laboratory infrastructure, shortages of skilled personnel, fragmented laboratory networks, and weak quality management systems (QMS). These limitations contribute to gaps in outbreak detection and response, quality assurance (QA), and workforce development [13].

Effective public health laboratory systems depend on both competent leadership and organizational, infrastructural, and operational capacities of the health system. While laboratory leadership development is crucial for building a resilient and sustainable workforce, it has traditionally received limited investment in many African contexts, despite substantial advances in pathogen genomic surveillance [15] and expanding support for workforce development [16–20]. Laboratory leaders are expected to manage complex diagnostic networks, uphold quality standards, maintain functional supply chains, and coordinate multisectoral responses during emergencies, often without formal preparation in organizational management, strategic planning, personnel supervision, or systems-level decision-making [21]. Also, laboratory-focused initiatives across the continent often struggle with structured leadership, and, in combination with the broader challenges affecting NPHIs and laboratories described above, some national laboratories continue to depend on externally managed programs. Consequently, even where technical investments have strengthened laboratory capacity, insufficient leadership and health system-wide limitations limit the ability of laboratories to sustain gains, achieve financial autonomy, and respond effectively during public health emergencies.

Evidence from across Africa indicates that strong leadership coupled with well-supported laboratory systems improves service quality, optimizes resource utilization, and strengthens workforce performance. In Zambia, for example, a structured leadership and management training program implemented between 2016 and 2018, which addressed the entire laboratory testing process, substantially improved ISO 15189 compliance, strengthened QMS, and advanced laboratories toward accreditation [21]. Similarly, leadership-driven quality improvement interventions in South Africa reduced unnecessary test utilization by up to 28% and generated sustained hospital-wide cost savings [22]. Conversely, promotion of technical laboratory staff to leadership roles without complementary leadership training can lead to weak delegation, communication gaps, and burnout, undermining outbreak readiness and operational continuity [23]. Effective public health laboratory leadership therefore requires competencies beyond technical proficiency, including communication, change management, cross-disciplinary coordination, and strategic prioritization. Programs such as the Global Laboratory Leadership Programme (GLLP) and the U.S. CDC Laboratory Leadership Service (LLS) support current and emerging leaders in integrating management practices [24], mentoring future leaders, and cultivating professional networks [23], while multisectoral engagement and academic integration have demonstrated potential for sustainable laboratory workforce development, as exemplified by Burkina Faso [24].

To address the gaps across Member States, Africa CDC, through the Africa Pathogen Genomics Initiative (Africa PGI) – DETECT Project (2024–2026), co-funded by the European Union, is investing in pathogen detection (i.e., molecular diagnostics and sequencing) and laying equal emphasis on leadership development as a core pillar of laboratory system strengthening. As part of this effort, a peer-to-peer laboratory leadership forum themed “Strengthening Laboratory Leadership, Systems, and Networks for Outbreak Detection in Africa” was convened under the coordination of Africa CDC. The forum aimed to strengthen Africa’s public health laboratory ecosystem by: (i) enhancing peer-to-peer exchange among laboratory leaders (ii) identifying cross-cutting challenges and innovative solutions, (iii) establishing a sustained community of practice to promote collaboration, and (iv) generating actionable recommendations.

In this article, we describe the experiences exchanged and lessons from the Africa PGI – DETECT Project, and strategies explored to strengthen diagnostic networks, optimize supply chains, improve outbreak preparedness, and mobilize sustainable resources, thereby positioning leadership at the center of resilient, genomics-enabled public health systems across Africa. The article is presented as a policy-oriented meeting report synthesizing participant experiences, country practices, and operational lessons generated during the workshop. It does not aim to provide empirical continental estimates but rather to identify priority implementation pathways for strengthening public health laboratory systems in Africa.

## Methods

### Workshop design and participants

The forum, conducted in Mombasa (Kenya), from 27 to 29 August 2025, brought together 24 heads of public health and reference laboratories, senior technical experts from 15 AU Member States (namely Botswana, Burkina Faso, Burundi, Cameroon, Union of Comoros, Democratic Republic of the Congo, Ethiopia, Malawi, Mali, Republic of the Congo, Senegal, South Africa, Tanzania, Uganda, and Zambia), and officials from Africa CDC. The three-day forum integrated expert-led presentations, country case studies, and interactive roundtable discussions structured around seven predefined thematic areas grouped under three overarching themes: (i) strengthening laboratory systems resilience (laboratory financing, sample referral systems, procurement and supply chains), (ii) laboratory operational excellence (equipment management, QMS, data systems), and (iii) resource mobilization (advocacy and sustainable financing mechanisms). Simultaneous English-French interpretation was provided to support full participation by both Anglophone and Francophone participants.

### Data collection and synthesis

Source material for this manuscript was derived from the official workshop report prepared by two designated rapporteurs using contemporaneous written notes from all sessions. Triangulation was undertaken through cross-validation between rapporteurs’ notes, participant presentations, and meeting discussions. Following the workshop, the draft report was circulated to all participants, including the wider community of Africa PGI – DETECT Project implementers, for review, revision, and approval to ensure factual accuracy and completeness. The present manuscript was subsequently developed from the validated workshop report. Published evidence is cited to contextualize workshop-derived observations, while workshop-derived insights are presented as participant experiences or consensus perspectives.

## Results and discussion

Workshop discussions highlighted multiple interrelated operational and structural challenges with diverse root causes (Table 1), which both influence and are influenced by the strength of public health laboratories and their leadership, the foundation of laboratory operations and long-term sustainability. Published evidence has documented many of these system constraints across public health laboratories in Africa [25–30]. Among these highlighted during our workshop, overreliance on donor funding for core laboratory functions emerged as a major system vulnerability to abrupt policy and financing shocks. The workshop provided operational and leadership-specific insights into how these challenges interact in practice and why their effects differ across countries.

**Table 1:** Summary of the major public health laboratory system challenges identified during the workshop, corresponding best practices, and proposed solutions

Key challenge identified (including root cause)	Best practices, proposed solutions, and way forward
**Overreliance on donor funding for core laboratory functions** (dependence on external aid, limited domestic investment, and absence of sustainability plans)	■ Integrate laboratory services into national health budgets ■ Develop sustainability and contingency frameworks ■ Diversify funding through public–private partnerships and philanthropic engagement ■ Reorient policy toward home-grown solutions ■ Empower local entrepreneurs
**Limited domestic financing and weak advocacy for sustainable laboratory systems** (weak evidence-based advocacy and low prioritization in national agendas)	■ Build advocacy using outbreak and surveillance data ■ Develop business models for laboratory revenue generation ■ Coordinate regional grant applications to enhance success rates
**Inefficient procurement, supply chain, and customs clearance systems** (fragmented national processes, weak governance structures, limited vendor presence, long clearance delays, ad hoc donations, short reagent shelf-life, and high servicing costs)	■ Establish strong governance frameworks and leadership mechanisms to enhance coordinated management of laboratory commodities and ensure uninterrupted service delivery ■ Institutionalize streamlined procurement and supply chain mechanisms ■ Adopt regional pooled procurement, demand aggregation, and digital customs systems ■ Strengthen integration with finance, regulatory agencies, and stakeholders ■ Establish clear national policies for equipment donations and product introduction ■ Implement forecasting tools and leverage regional distribution hubs ■ Coordinate management of reagents and equipment with digital monitoring systems
**Weak sample referral and transportation systems** (inadequate leadership engagement, absence of standardized logistics models and limited integration into national systems)	■ Ensure leadership commitment and coordinated implementation to support effective execution of sample transport systems ■ Develop, implement, and scale a unified, cost-effective sample transport framework to be used on the continent ■ Integrate courier and postal services within national laboratory logistics ■ Establish performance monitoring systems to evaluate timeline, quality of specimen, and cost
**High equipment downtime and unsustainable servicing models** (limited maintenance budgets, insufficient local technical capacity, and costly service contracts)	■ Establish regional service hubs ■ Train local technicians and engineers ■ Implement routine preventive maintenance schedules ■ Include service contracts in institutional budgets
**Gaps in laboratory quality management systems (QMS), controls, and accreditation** (limited training, funding constraints, and weak institutional commitment)	■ Institutionalize QMS mentorship and training programs ■ Promote regional quality hubs ■ Adopt stepwise accreditation processes aligned with national capacity
**Fragmented data management and poor interoperability** (manual data flow, weak integration between laboratory and surveillance systems, and inadequate cybersecurity measures)	■ Strengthen laboratory information management systems (LIMS) integration with national health information platforms ■ Promote digital transformation and data-sharing frameworks within a One Health approach ■ Adopt artificial intelligence (AI) tools to support data management and analysis workflows ■ Establish balance between sharing actionable insights versus data to enable rapid, evidence-based decisions
**Uneven decentralization of diagnostic capacity** (resource disparities between central and subnational laboratories and lack of sustainability planning)	■ Adopt phased, context-driven decentralization ■ Maintain hybrid diagnostic systems balancing central and regional functions (centralization versus decentralization) ■ Champion sustainability through domestic investment
**Limited workforce capacity and leadership development** (high attrition rates, inadequate mentorship, and insufficient leadership training)	■ Expand laboratory leadership and technical training programs ■ Promote peer-to-peer mentorship ■ Institutionalize continuous professional development
**Weak regional collaboration and fragmented knowledge exchange** (limited coordination and absence of structured platforms for information sharing)	■ Institutionalize and maintain national and regional laboratory networks ■ Establish annual symposiums for experience exchange ■ Promote biobanking and south-south collaboration

As summarized in Table 1, participants identified several best practices and proposed solutions, with responsibility for implementation shared among continental and regional institutions (including Africa CDC, as the continental public health agency, and in collaboration with WHO AFRO), national government bodies, laboratory leadership and management team (e.g., directors, quality managers, procurement officers), and industry and external partners.

### Theme 1: optimizing sample referral and supply chain networks for public health preparedness – day 1 (Wednesday, 27 August 2025)

#### Sub-theme 1.1. Impact of official development assistance (ODA) reduction on laboratory systems and outbreak detection in Africa

Recent reductions in ODA from the U.S. and Europe threaten to reverse decades of progress in laboratory systems, public health surveillance, outbreak detection, and biomedical innovations across sub-Saharan Africa [31–34]. The temporary suspension of U.S. government foreign assistance programs in January 2025 under the Trump administration brought into sharp focus the dependence of many African laboratories on external funding. Discussions during the forum, supported by a comparative analysis of recent ODA contractions, revealed widespread vulnerability across all participating countries, albeit with varying severity. A joint Africa CDC and Africa Society for Laboratory Medicine (ASLM) rapid assessment (unpublished data) showed that over half of the 30 surveyed countries relied on donor funding for core laboratory operations, with 50% unable to sustain services for more than three months without external support. Participants reported that the consequences of funding differed across countries, with several Member States reporting delays in reagent procurement, disruptions in equipment servicing, and challenges with sample transport systems, whereas only a few, specifically South Africa and Uganda, benefiting from stronger domestic financing, maintained greater operational continuity despite temporary funding lapses even during outbreaks. These comparative experiences suggest that domestic budget integration and institutionalized leadership structures are key determinants of laboratory system resilience (Table 1), as opposed to the episodic donor support often associated with outbreak response funding.

Evidence from the 2024 mpox outbreak, which affected more than 20 African countries, including the Democratic Republic of Congo, Malawi, Kenya, Sierra Leone, South Sudan, Tanzania, Uganda, and Zambia, illustrates deficiencies in health systems after external support was withdrawn. Following Africa CDC’s declaration of a continental public health emergency, funding suspensions in January 2025 led to immediate and substantial shortfalls across several countries, including Malawi (over US $350 million, 13% of its national budget), and disrupted HIV services, maternal health delivery, laboratory capacity, and surveillance systems [35]. Supporting their contributions, the participants from South Africa mentioned that similar disruptions were reported in their country, where abrupt reductions in funding from the President’s Emergency Plan for AIDS Relief (PEPFAR), U.S. Agency for International Development (USAID), and National Institutes of Health (NIH) led to clinic closures, significant workforce losses affecting over 8,000 health workers (approximately 53%), erosion of research capacity, and the suspension or winding down of critical HIV and tuberculosis research programs [34]. Comparable impacts have been reported in Uganda (over 2,000 health worker layoffs) and Nigeria [33]. Beyond health outcomes, the literature indicates that such retrenchments risk weakening innovation pipelines and altering strategic partnerships, with broader geopolitical implications for the region [31].

The forum participants pointed out that interim “stop-gap” support from Africa CDC and partners, such as the provision of diagnostics and essential equipment, helped sustain core laboratory functions during these disruptions. When complemented with domestic financing, these measures can buffer countries against acute shocks. Uganda’s experience during the 2025 Sudan EVD outbreak was highlighted as an illustration of rapid domestic reprioritization, where government-led resources reallocation stabilized laboratory operations and restored sample referral networks. The urgent need for increased domestic resource mobilization and diversified financing mechanisms, including engagement with African philanthropy and private-sector partnerships, came out strongly during the discussion (Table 1). The participants further noted that regional contingency planning, strategic reallocation of military expenditure, and the integration of essential diagnostics into national budgets could help maintain service continuity during disruptions, with financial sovereignty framed as a prerequisite for sustainable epidemic preparedness. Echoing Sarker et al. [36], this disruption is a wake-up call that AU Member States should leverage to catalyze domestic leadership, innovation, and durable health sovereignty across Africa. This call is urgent and requires a coordinated response, including an immediate audit of gaps in service provision, workforce, and systems created by the funding cut [34].

Published evidence has documented the effects of ODA withdrawal on health systems [31–36], but the workshop provided practical operational evidence of how countries absorb these shocks differently. A key finding from this workshop was the comparative observation that countries with stronger leadership advocacy and institutionalized domestic financing mechanisms recovered more rapidly and sustained operational continuity through some of the practices summarized in Table 1.

#### Sub-theme 1.2. Sample transportation and laboratory decentralization

Weak sample referral systems continue to delay diagnosis, compromise specimen quality, and divert skilled personnel away from clinical care and surveillance functions [37, 38]. Published evidence from Uganda, Burkina Faso, and Mali shows that coordinated hub-and-spoke and postal-based systems can substantially reduce turnaround times (TATs), improve biosafety, and expand diagnostic access when implemented at scale [37, 39]. Nonetheless, sustainability challenges, such as uneven infrastructure (e.g., different and ad hoc transport modalities across various disease surveillance systems), are still a concern. These findings were corroborated during the forum (Table 1), where participants described hybrid systems combining motorbike, postal, air, and maritime routes as the most common operational models across participating Member States. Participants noted that fully centralized systems often result in transport delays and overburdened national reference laboratories, while fully decentralized systems face sustainability challenges linked to infrastructure limitations, workforce shortages, and inconsistent quality systems. Hybrid models, where national reference laboratories retain advanced diagnostic functions while extending selected testing capabilities to subnational levels, were seen as the most feasible path forward. Digital enablement was viewed as foundational, with real-time sample tracking through mobile applications, laboratory information management systems (LIMS), and DHIS2 (formerly District Health Information Software) considered a critical enabler. Participants cautioned that mobile laboratories, although valuable for outreach in remote settings, remain costly and are largely limited to screening functions.

Scholarly data show that decentralizing laboratory diagnostics significantly increases testing coverage and reduces TAT by shortening specimen transit and accelerating molecular testing [40]. These findings support the rationale for phased decentralization supported by robust national reference laboratories. Based on both published evidence and country experience, Africa CDC recommended adopting a unified, hybrid, and cost-effective sample transportation model, such as Uganda’s hub-and-spoke system, which has demonstrated operational feasibility and cost-efficiency across multiple disease programs, to provide a unified framework for AU Member States, with implementation coordinated by ASLM. This model is intended to facilitate standardization, simplify coordination, and optimize resource use across countries, while maintaining flexibility to accommodate pathogen-specific requirements, country-specific operational settings, and differences in infrastructure and capacity. From the deliberations, it emerged that the effectiveness of this model is contingent on leadership commitment, coordinated implementation, and institutional integration across diagnostic and surveillance platforms.

#### Sub-theme 1.3. Procurement, supply chain, and customs clearance

Efficient procurement and supply chain systems have been shown to strengthen laboratory service delivery by minimizing stock-outs, improving inventory accuracy, and ensuring the timely availability of essential diagnostics and therapeutics [25, 26]. Using illustrative cases, participants identified procurement inefficiencies and customs delays across participating countries. For example, in one case, a five-month delay occurred between the purchase of sequencing reagents from an international supplier and their delivery to a laboratory in an African country, consistent with findings from studies documenting extended lead times for imported laboratory commodities in sub-Saharan Africa [27]. From the discussion, common challenges across sub-Saharan Africa include limited in-country vendor presence, prolonged customs clearance processes, and high servicing costs for imported equipment (Table 1).

Participants noted that poorly coordinated laboratory equipment donations and product introductions pose a critical challenge for laboratories participating Member States. They indicated that, while donations have expanded public health laboratory capacity, deficiencies such as incomplete documentation, lack of service agreements and spare parts, and limited training for maintenance personnel (predominantly leaving biomedical engineers without adequate instruction), make donated equipment difficult to install, maintain, or integrate into routine laboratory workflows. These observations mirror findings from prior studies on medical equipment donations in low-resource settings [41–43]. Participants added that sustainability is further constrained by the lack of long-term maintenance plans and by delays in customs clearance for donated equipment. In parallel, reagents for diagnostics and sequencing face distinct supply chain challenges, including short shelf-life, stringent storage requirements, and procurement processes that differ from those for biomedical equipment. Participants underscored that these barriers (Table 1) are compounded by weak governance structures, fragmented planning, poor coordination among procurement, finance, and technical departments. Therefore, strengthening leadership and governance may improve coordination of both reagents and equipment management, helping public health laboratories maintain continuous operations and prevent stock-outs or expired supplies [44].

Drawing on lessons from the COVID-19 pandemic and Uganda’s 2025 Sudan EVD outbreak, participants advocated for strengthening national procurement systems, closer integration with Ministries of Health and Finance, regional pooled procurement, pre-approved vendor frameworks, and digitalized customs clearance systems (Table 1). The forum noted the importance of establishing national policies and guidelines for equipment donation, including alignment with laboratory strategies, standardized platforms, and requirements for training, warranties, and post-sales support. Additional strategies identified, similar to regional experiences in South and Southeast Asia, included transparent and affordable pricing for genomic commodities from suppliers, alternative procurement models such as leasing or batch ordering, and national and regional forecasting tools to reduce purchasing lead times and costs [45]. Operationalization of these approaches would be supported by regional distribution hubs, harmonized customs and tax regimes, and robust post-sales maintenance and technical support structures, which are key enablers of timely access to genomic technologies and resilient laboratory supply systems [45].

Africa CDC’s Network Information Management System (NIMS: https://pginims.africacdc.org/about), currently in pilot phase, is a commended step and actionable priority toward streamlining equipment and reagent procurement, improving vendor accountability, and leveraging collective bargaining power across AU Member States. NIMS provides a centralized digital platform for managing laboratory equipment and inventory, with interactive dashboards that enhance visibility, track performance, and support efficient supply chain operations across participating laboratories. Integration of these digital capabilities could further reduce stock-outs, improve procurement planning, and strengthen accountability in the laboratory supply ecosystem.

### Theme 2: laboratory operational excellence for outbreak detection, characterization, and reporting – day 2 (Thursday, 28 August 2025)

#### Sub-theme 2.1. Equipment management and servicing

Africa CDC – Africa PGI and partners have supported AU Member States with state-of-the-art laboratory equipment, placing increased emphasis on maintenance and servicing requirements, which enable reliable diagnostics. However, participants concurred that high maintenance costs, limited technical expertise, and absence of routine preventive maintenance budgets continue to constrain equipment functionality across many public health laboratories (Table 1). Most laboratories reported equipment downtime due to delays in importing spare parts and unreliable power supply, and instruments that were obsolete, decommissioned, abandoned (often due to unaffordable service contracts and reliance on donor funding, which rarely covers maintenance), or had reached the end of their service life. Similar challenges have been reported in other resource-constrained settings, including Central Asia [28].

To address these challenges, the forum participants proposed several practical solutions, including establishing regional equipment servicing hubs, implementing routine preventive maintenance schedules, establishing mobile maintenance units, and developing technical training programs in collaboration with universities, technical institutes, and equipment and reagent manufacturers (Table 1). Consistent with published literature, forum participants underlined the importance of documented, QMS-aligned decommissioning procedures to safeguard equipment reassignment or disposal, preferably involving manufacturers or authorized service providers [28]. While some countries, such as Senegal, South Africa, Uganda, and Zambia, have established local service and maintenance services for smaller laboratory instruments, in-country capacity remains limited for sophisticated machines. A key insight from the forum was that equipment sustainability was identified as the principal challenge, with acquisition representing a less immediate concern, highlighting the need to prioritize long-term maintenance and service continuity. This leadership-focused programmatic finding extends the existing literature by shifting attention toward institutional maintenance planning. Actionable priorities include governments to institutionalize equipment servicing and maintenance budgets within national public health laboratory frameworks to ensure sustainability beyond donor-funded cycles and protect prior investments in laboratory infrastructure (Table 1).

#### Sub-theme 2.2. Laboratory QMS, quality controls, certification, and accreditation

The implementation of a robust laboratory QMS [46], including standardized workflows [47], is the cornerstone for generating high-quality test results [29, 48]. Across Africa, laboratory QMS has seen significant, though uneven, improvement efforts and progress [49–51], mainly driven by structured programs [52]. Despite these advances, implementation and maintenance of QMS continue to experience challenges [29, 30], such as high costs associated with external quality assessment (EQA), weak equipment maintenance systems, shortages of trained personnel, and fragmented governance structures [28–30, 53, 54]. Drawing from our roundtable discussion, such challenges in QMS, including bioinformatics, which has received little attention, are due to the complexity of laboratory processes, the need for workflow standardization, and gaps in workforce expertise (Table 1). Therefore, participants reaffirmed that public health laboratories must prioritize the twelve current quality system essentials (QSEs), such as personnel, equipment, process management, and documents and records, as core frameworks for developing and implementing a QMS [49, 55], following practical guidance, including recommendations specific to laboratories performing next-generation sequencing [55].

Participants unanimously agreed that QMS must be operationalized in daily laboratory operations rather than treated as a documentation exercise. This position is supported by evidence that a well-implemented QMS, with policies, processes, and procedures structured around QSEs, significantly improves analytical accuracy, patient safety, and clinical outcomes, particularly during the introduction of complex tests [29, 50, 54, 56, 57]. Accordingly, QA activities, EQA, and inter-laboratory comparisons were identified as integral to supporting the credibility of laboratory outputs. Countries with established laboratory capacity, such as Senegal through its West African Regional Initiative for Laboratories (WARIL), shared successful models of regional EQA panel production to help offset costs. Africa CDC and ASLM were commended for their ongoing efforts to provide mentorship and training in QMS (e.g., in genomics and bioinformatics), including through the Strengthening Laboratory Management Toward Accreditation (SLMTA) and Stepwise Laboratory Quality Improvement Process Towards Accreditation (SLIPTA) frameworks, as well as through centralized coordination of EQA activities for molecular testing assays. However, implementation of QMS in genomics and bioinformatics remains comparatively under-addressed. Participants advocated for stepwise accreditation aligned with local capacity and supported by regional quality hubs. These measures, together with leadership commitment and resource availability, are needed for sustainable quality improvement [53, 57]. Consequently, participants called for coordinated training programs, leadership development initiatives, and dedicated financing mechanisms to institutionalize laboratory QMS across Member States, including the adoption of stepwise accreditation approaches to support progressive quality improvement and sustainable accreditation readiness.

#### Sub-theme 2.3. Data management, reporting, and dissemination

Fragmented, non-interoperable data systems remain a major challenge for public health laboratories across participating Member States (Table 1). The workshop contextualized data interoperability within One Health and data sovereignty frameworks, recognizing its role in strengthening integrated surveillance systems and national data stewardship. The discourse highlighted that countries such as Senegal, South Africa, Tanzania, and Zambia have integrated LIMS with DHIS2 to support national reporting, while others continue to rely on paper-based systems or hybrid approaches. For example, Malawi reported recent mpox results using a combination of electronic and manual data transmission. The representatives from the Union of Comoros described inter-island data transmission difficulties between Grande Comore, Anjouan, and Mohéli. In South Africa, LIMS-enabled data collection is well established; however, instances of cyberattacks [58], internet instability, and power outages have been reported. Meanwhile, difficulties in consolidating data from multiple partner-funded projects were reported in the Democratic Republic of Congo. Participants recognized the importance of timely, validated data and insight sharing during outbreaks, particularly for pathogen genomic surveillance. They also highlighted the need for high-quality data accompanied by appropriate contextual metadata, adherence to ethical and privacy standards, and the use of sovereignty-preserving platforms that enable the secure sharing of actionable insights while maintaining national ownership of data, as proposed in continental efforts to establish a federated African pathogen genomics data-sharing system, the Africa Genome Archiving for Response and Insights (AGARI) platform [59].

Consistent with previous studies [60], participants reiterated the importance of high-quality standards for data integrity, decision-making, and public health interventions. They also emphasized that genomic data analysis must ensure that data generated, shared with public health authorities, and submitted to databases are accurate, reliable, traceable, repeatable, and reproducible, in line with established recommendations [61]. Despite these priorities, substantial barriers continue to limit timely, interoperable genomic and public health data sharing. These include data security challenges, limited digital infrastructure, and the lack of integrated One Health data systems linking human, animal, and environmental surveillance (Table 1). Participants identified several promising practices to strengthen data management, reporting, and dissemination. These include dashboards, real-time LIMS tracking, standardized reporting templates, leadership support for data governance, artificial intelligence-powered platforms, collaborative research initiatives, and routine dissemination of results to stakeholders through regular coordination meetings.

### Theme 3: resource mobilization, reporting, and publication – day 3 (Friday, 29 August 2025)

#### Sub-theme 3.1. Advocacy, financing, and sustainable funding

Participants highlighted challenges in securing and sustaining resources for their public health laboratories. While countries such as South Africa and Uganda benefit from comparatively higher levels of government funding, many Member States, including the Union of Comoros, remain heavily dependent on international donor support. Even in better-resourced settings, funding constraints are evident. For example, the participant from Uganda stated that the country was compelled to rapidly mobilize supplementary resources during the COVID-19 pandemic, while the representatives from South Africa indicated the importance of donor aid in targeted initiatives such as AMR surveillance. Although outbreaks are disruptive and pose significant operational challenges, they also create opportunities to demonstrate the value of laboratory systems and mobilize political and financial support. Public health institutions in countries including South Africa and Senegal mentioned successfully leveraging outbreak response activities to secure increased national allocations and establishing fee-based sequencing services designed to recover costs while supporting public health priorities. Participants observed that more efficient and resilient laboratory systems were characterized not only by larger budgets, but also by stronger leadership advocacy and institutional planning. They further agreed that advocacy and financing efforts must be evidence-based.

To improve grant access to funding and promote sustainability, participants proposed pooling project resources across institutions, engaging multiple international donors, and strengthening domestic financing mechanisms. There was strong consensus that Africa CDC could play a more prominent role in coordinating grant applications, facilitating aggregated demand and pooled procurement, supporting biobanking platforms, and promoting cross-country collaboration. Participants also noted that public–private partnerships and African philanthropic funding remain underutilized and should be prioritized in future engagement strategies. Finally, long-term institutional commitment, policy support, investment, and leadership were identified as key factors to unlocking advocacy, financing, and sustainable funding for resilient health systems in Africa. The workshop showed the pathways through which leadership-led advocacy can translate outbreak evidence into sustainable financing opportunities for public health laboratory systems.

#### Key lessons, way forward, and next steps

Despite notable progress, laboratory capacity across Africa remains heterogeneous and uneven, with measurable variability in testing capacity among AU Member States [62]. Taken together, the challenges identified in this workshop, those related to leadership, present opportunities for transformation. Workshop discussions showed that countries with more resilient laboratory systems (e.g., South Africa, Uganda) were characterized less by technology availability and more by stronger domestic financing, structured leadership, and institutionalized systems. There is a need to invest in public health laboratories at the national level to achieve long-term sustainability and resilience, strengthen leadership and laboratory capacities within Member States, and reduce dependence on external or regional support.

Across all themes (including sub-themes), eight cross-cutting priorities emerged: (i) institutionalizing sustainable domestic financing for public health laboratory systems, including collaboration between Ministries of Health and Finance, (ii) optimizing supply chain and sample-referral logistics, including addressing customs clearance procedures for biomedical equipment and reagents, (iii) establishing sustainable equipment servicing and maintenance capacity, (iv) strengthening QMS, (v) improving data interoperability within a One Health framework, (vi) adopting phased, context-appropriate diagnostic decentralization, (vii) enhancing workforce capacity and leadership development, and (viii) strengthening national and regional laboratory systems and laboratory leadership networks through collaboration, institutionalizing peer-to-peer leadership exchange, and leveraging operational capacity. These findings reflect implementation experiences from participating Member States and should be interpreted as policy-relevant lessons rather than as generalizable continental evidence. Participants concluded with a set of practical next steps to guide future action. These include mapping national laboratory financing landscapes and gathering data to quantify the challenges described, developing and implementing a unified cost-effective sample transport model adaptable across Member States, maintaining knowledge exchange through regular regional symposia, and systematically documenting discussions, lessons learned, and best practices to inform policy, guide implementation, and preserve institutional memory.

## Conclusions

Public health laboratory capacity across Africa has expanded significantly, but leadership development and system institutionalization have not advanced similarly. The Africa PGI – DETECT Project Laboratory Leadership Workshop demonstrated that resilient public health laboratory systems require more than infrastructure. Immediate priorities emerged, including integrating laboratory financing into domestic budgets to reduce donor dependency, institutionalizing equipment servicing and QMS as core operational functions, and strengthening interoperable laboratory and surveillance data systems under coordinated leadership frameworks. In order to sustain these gains across Africa, particularly in the participating Member States, the network of laboratory leaders established through the workshop should be maintained and expanded, with a clear framework to support their continued collaboration and exchange of knowledge. A key contribution of the workshop was not the identification of new laboratory challenges, many of which are already well recognized, but the clarification of leadership-driven operational pathways for implementation, coordination, and sustainability. The message from the workshop is unequivocal: Africa must strengthen laboratory leadership, resolve operational and structural gaps, assume full ownership of its public health laboratory systems, and chart its own path toward sustainable health security.

## Data Availability

Not applicable.

## Abbreviations

Africa CDC: Africa Centres for Disease Control and Prevention
Africa PGI: Africa Pathogen Genomics Initiative
AGARI: Africa Genome Archiving for Response and Insights
AI: Artificial Intelligence
AMR: Antimicrobial Resistance
ASLM: Africa Society for Laboratory Medicine
AU: African Union
COVID-19: Coronavirus Disease 2019
DHIS2: District Health Information Software 2
EQA: External Quality Assessment
EVD: Ebola Virus Disease
GLLP: Global Laboratory Leadership Programme
HIV: Human Immunodeficiency Virus
ISO: International Organization for Standardization
LIMS: Laboratory Information Management System
LLS: Laboratory Leadership Service
NIH: National Institutes of Health
NIMS: Network Information Management System
NPHI: National Public Health Institute
ODA: Official Development Assistance
PEPFAR: President’s Emergency Plan for AIDS Relief
QA: Quality Assurance
QMS: Quality Management Systems
QSEs: Quality System Essentials
SLIPTA: Stepwise Laboratory Quality Improvement Process Towards Accreditation
SLMTA: Strengthening Laboratory Management Toward Accreditation
TAT: Turnaround Time
U.S. CDC: United States Centers for Disease Control and Prevention
USAID: United States Agency for International Development
WARIL: West African Regional Initiative for Laboratories
WHO AFRO: World Health Organization Regional Office for Africa
